# *ALDH2* polymorphism rs671 is a predictor of PD-1/PD-L1 inhibitor efficacy against thoracic malignancies

**DOI:** 10.1186/s12885-021-08329-y

**Published:** 2021-05-22

**Authors:** Akiko Matsumoto, Chiho Nakashima, Shinya Kimura, Eizaburo Sueoka, Naoko Aragane

**Affiliations:** 1 Department of Social Medicine, Saga University School of Medicine, 5-1-1 Nabeshima, Saga, 849-8501 Japan; 2 Division of Hematology, Respiratory Medicine and Oncology, Saga University School of Medicine, Saga, Japan; 3 Department of Clinical Laboratory, Saga University Hospital, Saga, Japan

**Keywords:** ALDH2, rs671, Immune checkpoint inhibitors, Thoracic malignancy

## Abstract

**Background:**

Aldehyde dehydrogenase 2 (ALDH2) plays an important role in the endogenous aldehyde detoxification of various types of cells. ALDH2*2, a variant allele of the *ALDH2* polymorphism rs671, leads to decreased enzymatic activity. ALDH2*2 may enhance tumor antigen presentation due to aldehyde-induced DNA damage while suppressing peripheral blood T cell counts and T cell activation.

**Methods:**

On the basis of our hypothesis that rs671 affects the sensitivity of immune checkpoint inhibitors (ICIs), we evaluated the effects of rs671 on patients with thoracic malignancies who started ICI therapy in 2016–2019. The cohort consisted of 105 cases, including 64 cases with adenocarcinoma and 30 cases with squamous cell carcinoma, 49 of whom were ALDH2*2 carriers. The first ICI was PD-1/PD-L1 inhibitor (Nivolumab, Pembrolizumab, or Atezolizumab) in all cases.

**Results:**

The best response to anti-PD-1/PD-L1 therapy (partial response/stable disease/progressive disease) was 36%/50%/14% in the rs671(−) cases; however, the response was relatively poor in the rs671(+) cases (27%/29%/45%, respectively) (*p* = 0.002). The hazard ratio (95% confidence interval) of disease progression within the observation period of 6 months for the rs671(+) cases was estimated to be 5.0 (2.5–10) after the adjustment for covariates, including sex, Brinkman index, treatment line, tumor tissue programmed death-ligand 1 positivity rate, tumor tissue *EGFR* mutation. This association was also maintained in a stratified analysis, suggesting that ALDH2*2 is an independent negative predictive factor for the short-term prognosis of anti-PD-1/PD-L1 therapy. Thus, the progression-free survival (PFS) ratio of the rs671(+) cases decreased rapidly after ICI initiation but was eventually higher than that of the rs671(−) cases (restricted mean survival time in 12 months from 2 to 3 years afterward was 1.3 times that of the rs671(−) cases). Moreover, the highest PFS ratio after 2 years among sub-groups was found in the first-line treatment sub-group of rs671(+) group (40%).

**Conclusions:**

Our study suggests that rs671 may be an accurate and cost-effective predictor of PD-1/PD-L1 inhibitor treatment, in which optimal case selection is an important issue.

**Supplementary Information:**

The online version contains supplementary material available at 10.1186/s12885-021-08329-y.

## Background

Aldehyde dehydrogenase 2 (ALDH2) is expressed in many tissues, including blood cells [1, 2], and metabolizes endogenous aldehydes, such as formaldehyde, acetaldehyde, and 4 hydroxynonenal (4HNE) [3, 4]. Approximately half of the Japanese population and at least 2% of the global population shows the low-activity phenotype derived from the *ALDH2* genetic polymorphism rs671 (the variant allele is named ALDH2*2), which is associated with differences in lifestyle habits, disease risks, and drug sensitivities [5, 6]. The association is complicated, bidirectional, and rather strong [7]. For example, esophageal cancer is less common among ALDH2*2 carriers due to reduced drinking habits, but ALDH2*2 carriers with drinking habits show the highest risk because of accumulated aldehydes [8]. Additionally, ALDH2*2 is reported to increase the risk for leprosy [9], whereas viral hepatitis is mild in ALDH2*2 carriers [10], likely due to the alleviation of inflammation by the presence of aldehydes [11, 12]. Because hepatitis is a primary carcinogenesis promoter, it is reasonable that ALDH2*2 is reported as a protective factor against liver cancer [13, 14].

Immune checkpoint inhibitors (ICIs) are an innovative cancer treatment that provides benefits for some but not the majority of patients; therefore, understanding the ICI-sensitive population is an important challenge. To date, rs671 has not been studied as a potential predictor of ICI treatment, but it may have a complicated, bidirectional, and strong effect on ICI therapy for the following reasons: 1) Cancer cells of ALDH2*2 carriers may show more DNA damage induced by aldehyde exposure during smoking and drinking [15, 16], resulting in an increased presentation of antigens to immune cells, which is advantageous in ICI treatment. 2) Because endogenous 4HNE, a typical endogenous aldehyde that accumulates in ALDH2*2 carriers, delays cell proliferation [3, 17–20], ICI resistance due to genetic mutations in cancer cells [21, 22] is less likely to occur. 3) However, high aldehyde concentrations can suppress immune cell activation [12], making the short-term effect of ICIs difficult to detect. 4) Nevertheless, T cell exhaustion is unlikely to occur [23, 24], and this may be advantageous in long-term ICI therapy. 5) Lastly, the low T cell count in the peripheral blood of ALDH2*2 carriers reported previously may have a negative effect on ICI treatment [25]. Thus, to verify the hypothesis that ALDH2*2 carriers show a different ICI sensitivity compared with non-carriers, we investigated patients with ICI-treated thoracic malignancies.

## Methods

### Patients

The subjects were 106 patients with thoracic malignancies who received ICI treatment at the Division of Hematology, Respiratory Medicine and Oncology, Saga University School of Medicine from February 2016 to May 2019 and provided written consent for the study including genetic analyses (all patients were invited and all agreed). There was no restriction on the number of ICI doses, type of ICI, and chemotherapy after the first ICI dose. We obtained relevant information from the electronic medical records. The *ALDH2* genotype (rs671) was determined in DNA extracted with DirectPCR Lysis Reagent (Viagen Biotech, Inc. Los Angeles, CA) form peripheral blood mononuclear cells stored at − 20 °C using a TaqMan® SNP genotyping assay system in accordance with the instructions (ThermoFisher Scientific, Waltham, MA, USA). One patient was excluded from the study after less than 3 months of observation without disease progression. The study was approved by the clinical study ethics review committee of Saga University (project ID R1–16) and conducted accordingly.

### Statistics

#### Main outcomes: best response to ICI therapy

One of the main outcomes was the best response to ICI treatment. Best responses were classified as Complete Response (CR), Partial Response (PR), Stable Disease (SD), and Progressive Disease (PD) according to RECIST Ver1.1 [26]. CR is defined as the disappearance of all target lesions with any pathological lymph nodes reduced in the short axis to < 10 mm and PR as at least a 30% decrease in the sum of target lesion diameters, taking as reference the baseline sum diameters. PD is defined as at least a 20% increase in the sum of target lesion diameters, taking as reference the smallest sum, and the sum must also demonstrate an absolute increase of at least 5 mm. Lastly, SD is defined as neither sufficient shrinkage to qualify for PR nor sufficient increase to qualify for PD, taking as reference the smallest sum diameters.

#### Main outcomes: restricted mean survival time (RMST)

**Fig. 1:**
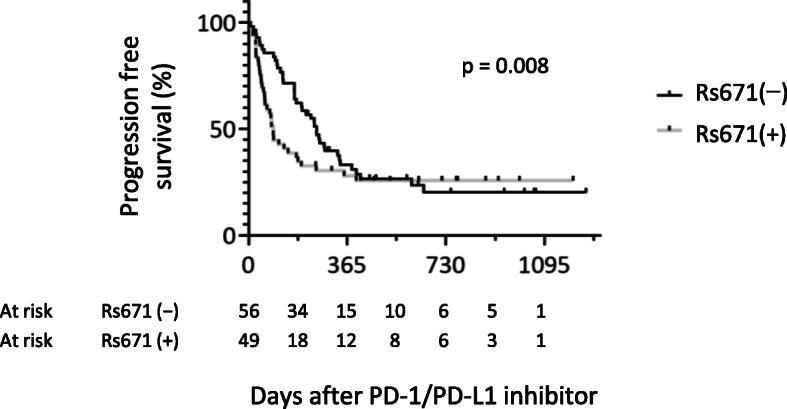
Progression-free survival after the initiation of PD-1/PDL-1 inhibitor therapy. Kaplan–Meier plots were shown for patients with a thoracic malignancy. ICI, immune checkpoint inhibitor; Rs671(−), ALDH2*1/*1 (*n* = 56), rs671(+); ALDH2*1/*2 or ALDH2*2/*2 (*n* = 49). p, p value for Gahan–Breslow–Wilcoxon test

The RMST introduced by Royston and Parmar [27] of the progression-free survival (PFS) was used because a proportional hazard assumption has not been established between the rs671 groups for PFS (Figs. 1, S1). RSMT was estimated as the area under the survival curve between the time points (LIFETEST procedure in SAS 9.4, SAS Institute Inc., Cary, NC, USA).

#### Secondary outcomes: PFS ratio during a 6-month observation period

From the biological background described in the Background section, the effect of ICIs on short-term prognosis and long-term prognosis may differ for each rs671 group. From the report on the adhesion time of programmed cell death-1 (PD-1) antibodies and memory T cells^21^, the half-life of PD-1 inhibitor is estimated to be several months. The longer the observation period, the more effects of time-dependent covariates that cannot be adjusted; for example, the number of ICI administrations after the second dose (up to 68 times in this study) (Table S1), drug used, presence or absence of adverse reactions, and onset time of adverse reactions. Thus, we aimed to directly compare the short-term effects of the initial ICI by limiting the observation period. Because proportional hazard assumption during this period is established (Fig. S1), a Cox proportional hazard model was used to estimate the hazard ratio (HR) after multivariate adjustment (PHREG procedure in SAS 9.4). Covariates were identified as attributes suspected to be associated with disease progression or rs671. For example, *EGFR* mutations in tumor tissue, female sex, non-smoker, and adenocarcinoma tissues are predictors of poor prognosis [28–30], and *EGFR* mutations and programmed death-ligand 1 (PD-L1) expression are associated with smoking habits [30, 31]. Smoking habit is also associated with rs671 [32, 33]. Neutrophils dominate the immune cell composition in non-small cell lung cancer (NSCLC) [34] and its infiltration into cancer tissue is reported to associate with prognostic risk score in squamous cell carcinoma [35]. Thus, sex, age (continuous), Brinkman index (< 100, < 1000, ≥1000) (ordinal), type of first ICI, tumor histotype, TNM classification (categorical), number of lines (first, second, third, and later) (categorical), chemistry with ICIs, PD-L1 positivity ratio (< 1, < 50, − 100%, unassessed) (categorical), *EGFR* mutation ((+), (−), unassessed) (categorical), neutrophil count in peripheral blood (log scale), and lymphocyte count in peripheral blood (log scale) were set as the covariates. Additionally, as a time-dependent covariate, the presence or absence of immune-related adverse events (irAEs) (defined as ICI withdrawal or prednisolone administration due to immune-related side effects) that occurred prior to disease progression was used. The number of days before the appearance of irAEs was entered as a continuous variable (Supporting information, SAS code). Chemotherapy, which started before disease progression, was also considered. However, because it was applied to only one case, it was not used as a variable. In addition, stratified analyses were performed in case the multivariate adjustment was inadequate.

#### PFS ratio per rs671 group

**Fig. 2:**
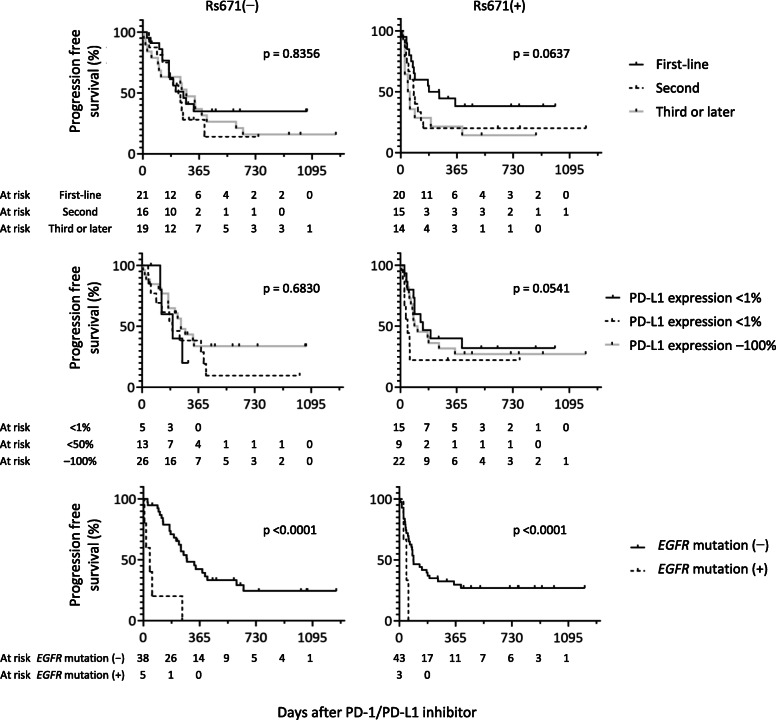
Progression-free survival after the initiation of immune checkpoint inhibitor therapy by *ALDH2* rs671 polymorphism. Kaplan–Meier plots were shown for patients with thoracic malignancies. ICI; immune checkpoint inhibitor, Rs671(−); ALDH2*1/*1, rs671(+); ALDH2*1/*2 or ALDH2*2/*2. p, *p* value for Gahan–Breslow–Wilcoxon test

The PFS ratio was determined by rs671 groups because the effects of covariates on ICI treatment are likely to differ between rs671 groups based on biological assumptions. For example, chemotherapy before ICI for patients with rs671(+) may cause more T cell immunity loss because their basic T cell count is lower than that of patients with rs671(−) [25]. If grouped by rs671, the proportional hazard assumption for the total observation period is maintained between sub-groups (Fig. 2). Therefore, the HR was estimated using a Cox proportional hazard model (PHREG procedure in SAS 9.4).

## Results

### Basic characteristics

The characteristics of the patients are shown in Table 1. All patients had anti-PD-1/PD-L1 therapy for the first dose. The distribution of tumor tissue PD-L1 positivity rates was different between the *ALDH2* genotypes. In the time-dependent variables shown in Table S1, the number of ICI administrations was low in the rs671(+) group.

**Table 1:** Characteristics of patients by *ALDH2* genotype

	Total		Rs671(−)		Rs671(+)		p
All	105		56		49		
Sex							
Male	89	(85%)	44	(79%)	45	(92%)	0.059
Female	16	(15%)	12	(21%)	4	(8%)	
Age (years)							
Median (IQR)	69		69		69		0.835
40–49	6	(6%)	4	(7%)	2	(4%)	0.970
50–59	12	(11%)	6	(11%)	6	(12%)	
60–69	37	(35%)	20	(36%)	17	(35%)	
70–79	46	(44%)	24	(43%)	22	(45%)	
80–89	4	(4%)	2	(4%)	2	(4%)	
Brinkman Index							
< 100	14	(13%)	10	(18%)	4	(8%)	0.330
< 1000	39	(37%)	19	(34%)	20	(41%)	
≥ 1000	52	(50%)	27	(48%)	25	(51%)	
Type of ICI (first dose)							
Pembrolizumab	45	(43%)	24	(43%)	21	(43%)	0.658
Atezolizumab	20	(19%)	9	(16%)	11	(22%)	
Nivolumab	40	(38%)	23	(41%)	17	(35%)	
Tumor histotype							
Squamous cell carcinoma	30	(29%)	19	(34%)	11	(22%)	0.176
Adenocarcinoma	64	(61%)	31	(55%)	33	(67%)	
Pleomorphic carcinoma	5	(5%)	1	(2%)	4	(8%)	
Mesothelioma	2	(2%)	2	(4%)	0	(0%)	
Other^a^	4	(4%)	3	(5%)	1	(2%)	
TNM classification							
Stage III	25	(24%)	15	(27%)	10	(20%)	0.148
Stage IV	51	(49%)	30	(54%)	21	(43%)	
Unknown	29	(28%)	11	(20%)	18	(37%)	
Treatment line							
First-line	41	(39%)	21	(38%)	20	(41%)	0.840
Second-line	31	(30%)	16	(29%)	15	(31%)	
Third-line and later	33	(31%)	19	(34%)	14	(29%)	
Chemotherapy with first ICI							
No	99	(94%)	52	(93%)	47	(96%)	0.683
Yes	6	(6%)	4	(7%)	2	(4%)	
PD-L1 (+) ratio in cancer tissue							
< 1%	20	(19%)	5	(9%)	15	(31%)	0.012
< 50%	22	(21%)	13	(23%)	9	(18%)	
≥ 50%	48	(46%)	26	(46%)	22	(45%)	
Unassessed	15	(14%)	12	(21%)	3	(6%)	
*EGFR* mutation in cancer tissue							
(−)	81	(77%)	38	(68%)	43	(88%)	0.400
(+)	8	(8%)	5	(9%)	3	(6%)	
Unassessed	16	(15%)	13	(23%)	3	(6%)	
Neutrophil count in peripheral blood							
Geometric mean (GSD) (/μL)	4663	(3134)	4501	(5167)	4854	(4808)	0.456
< 3500/μL	29	(28%)	19	(34%)	10	(20%)	0.273
< 5000/μL	37	(35%)	19	(34%)	18	(37%)	
≥ 5000/μL	39	(37%)	18	(32%)	21	(43%)	
Lymphocyte count in peripheral blood							
Geometric mean (GSD) (/μL)	1240	(752)	1141	(1093)	1362	(1344)	0.056
< 1000/μL	31	(30%)	19	(34%)	12	(24%)	0.151
< 1500/μL	39	(37%)	23	(41%)	16	(33%)	
≥ 1500/μL	35	(33%)	14	(25%)	21	(43%)	

Rs671(−); ALDH2*1/*1, rs671(+); ALDH2*1/*2 or ALDH2*2/*2, *ICI* Immune checkpoint inhibitor, *IQR* Interquartile range, *PD-L1* Programmed death-ligand 1, *EGFR* Epidermal growth factor receptor. ^a^ includes combined small cell lung carcinoma, adenosquamous carcinoma of the lung, and non-small-cell lung cancer-not otherwise specified. *GSD* Geometric standard deviation. *p* Probability value for Chi-squared test, Fisher’s exact test, Wilcoxon rank-sum test, or unpaired t-test

### Best response and RMST

The best response to anti-PD-1/PD-L1 therapy is shown in Table 2. In all cases, the best effect was observed at the initial ICI evaluation, and the response was better in the rs671(−) group than in the rs671(+) group (Spearman’s rank correlation coefficient = 0.27, *N* = 105, *p* = 0.008). The same analysis limited to 103 patients with NSCLC (excluding patients with mesothelioma) obtained similar result (Table S2, Spearman’s rank correlation coefficient = 0.27, *N* = 103, *p* = 0.007). The PFS curve (Fig. 1) also suggested that ALDH2*2 has a negative effect early after PD-1/PD-L1 inhibitor initiation, but after 2 years, the PFS ratio was higher in the rs671(+) group than in the rs671(−) group, and after 2–3 years (12-month period), the RMST was 0.26 in the rs671(+) group and 0.20 in the rs671(−) group (Table 3). Similar results were obtained for patients with NSCLC (Table S3).

**Table 2:** Overall best response per RECIST Ver1.1. by *ALDH2* genotype

	Total		Rs671(−)		Rs671(+)		p
Best response to immune checkpoint inhibitor							
Complete response	0	(0%)	0	(0%)	0	(0%)	0.0022
Partial response	33	(31%)	20	(36%)	13	(27%)	
Stable disease	42	(40%)	28	(50%)	14	(29%)	
Progressive disease	30	(29%)	8	(14%)	22	(45%)	
Disease control rate	71%		86%		55%		0.0005

*N* = 105. Rs671(−); ALDH2*1/*1, rs671(+); ALDH2*1/*2 or ALDH2*2/*2, disease control rate; (all − progressive disease)/all, *p* probability value for Chi-squared test

**Table 3:** Progression-free survival rate after the initiation of immune checkpoint inhibitors

Observation period	Restricted mean survival time	
	Rs671(−)	Rs671(+)
0–6 months	0.82	0.58
6–12 months	0.46	0.31
12–24 months	0.25	0.26
24–36 months	0.20	0.26

Rs671(−); ALDH2*1/*1, rs671(+); ALDH2*1/*2 or ALDH2*2/*2

### Multivariate-adjusted HR during a 6-month observation period

**Fig. 3:**
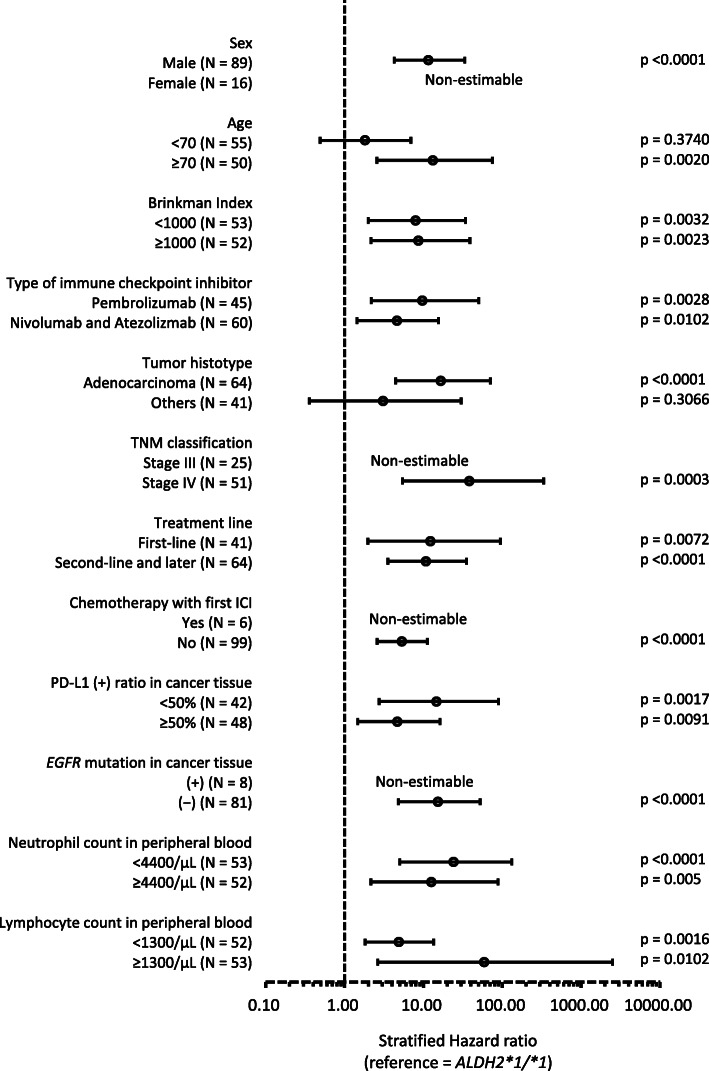
Stratified hazard ratio of cancer progression for ALDH2*2 carriers estimated from a 6-month observation. Reference = ALDH2*1/*1 carriers. Hazard ratio and 95% confidence interval (error bar) were estimated by Cox proportional hazard model adjusted for covariates used in Model 4 in Table 4. PD-L1; programmed death-ligand 1, *EGFR*; epidermal growth factor receptor. Cases with unknown TNM classification (*N* = 29), unknown PD-L1 (+) ratio in cancer tissue (*N* = 15), and unknown *EGFR* mutation in cancer tissue (*N* = 16) were excluded

The multivariate-adjusted HR during a 6-month observation period is shown in Table 4 and Table S4. In model 4 with all variables, the HR (95% confidence interval (CI), *p*-value) of the rs671(+) group was estimated to be 5.4 (2.7–11, *p* < 0.0001). It was 4.5 (2.2–9.2, p < 0.0001) (Akaike’s Information Criterion = 462) and almost unchanged after the adjustment of the time-dependent variables (the presence or absence of irAEs and timing of onset). In the stratified analysis, the HR was estimated to be high in the rs671(+) group almost consistently (Fig. 3). As a result of the same calculation for overall mortality, the same tendency was shown, although the estimation accuracy was largely disturbed (Fig. S2, Tables S5 and S6).

**Table 4:** Hazard ratio of cancer progression for ALDH2*2 carriers estimated from a 6-month observation

	Rs671	HR	95% CI	p	AIC
Model 1	(−)	1.00	(reference)		464
	(+)	3.33	(1.80–6.15)	0.0001	
Model 2	(−)	1.00	(reference)		465
	(+)	4.89	(2.37–10.1)	< 0.0001	
Model 3	(−)	1.00	(reference)		460
	(+)	5.04	(2.48–10.2)	< 0.0001	
Model 4	(−)	1.00	(reference)		444
	(+)	5.42	(2.65–11.1)	< 0.0001	

N = 105. Rs671(−); ALDH2*1/*1, rs671(+); ALDH2*1/*2 or ALDH2*2/*2, HR Hazard ratio by Cox proportional hazard model, *CI* Confidence interval, *AIC* Akaike’s Information Criterion

Model 1: adjusted for sex, age (continuous), Brinkman Index (< 100, < 1000, ≥1000) (ordinal), type of first immune checkpoint inhibitor (ICI), tumor histotype, TNM classification (categorical), number of lines (first, second, third, and later) (categorical), and chemotherapy with ICI

Model 2: adjusted for the covariates in model 1 and the PD-L1 positivity ratio (< 1, < 50%, ≥50%, unassessed)

Model 3: adjusted for the covariates in model 2 and *EGFR* mutation ((+), (−), unassessed)

Model 4: adjusted for the covariates in model 3, log (neutrophil count in peripheral blood) and log (lymphocyte count in peripheral blood)

### Association between PFS and the other variables by rs671 groups

**Fig. 4:**
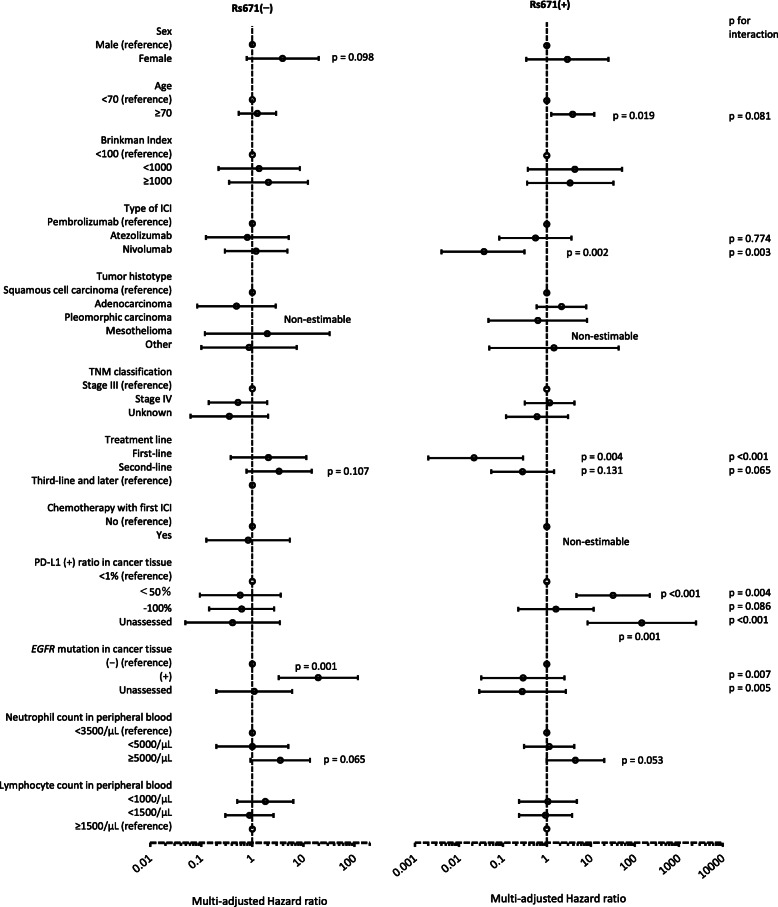
Hazard ratio of cancer progression by *ALDH2* genotype estimated from the entire observation period. Rs671(−); ALDH2*1/*1, rs671(+); ALDH2*1/*2 or ALDH2*2/*2. Hazard ratio and 95% confidence interval (error bar) was estimated by the Cox proportional hazard model includes all explanatory covariates shown in this figure. ICI; immune checkpoint inhibitor, PD-L1; programmed death-ligand 1, *EGFR*; epidermal growth factor receptor, *p*-value for interactions was estimated by the model including all covariates shown above, rs671, and interaction in terms of sex*rs671, type of ICI*rs671, treatment line*rs671, PD-L1 ratio*rs671, and *EGFR* mutation*rs671. *P* values are shown separately for each genotype with forest plot if *p* < 0.2. All *p* values for interaction are shown on the right

The PFS curve is shown in Fig. 2, and the multivariate-adjusted HR is shown in Fig. 4. The multivariate-adjusted HR of PFS showed significant association with age, type of ICI, treatment line, PD-L1 antibody positivity rate, and *EGFR* mutation for either type of rs671; an interactive association with rs671 was suggested for type of ICI, treatment line, PD-L1 antibody positivity rate, and *EGFR* mutation based on the interaction analysis (Fig. 4). The treatment line was associated with PFS only in the rs671(+) group, and the first-line group showed the best treatment outcome. The PD-L1 positivity rate was also associated with PFS only in the rs671(+) group; however, there was no dose-response relationship (a middle level was associated with the highest HR). Only the rs671(−) group showed short PFS among the groups with *EGFR* mutations.

## Discussion

As expected, the *ALDH2* polymorphism rs671 influenced the effects of anti-PD-1/PD-L1 therapy on thoracic malignancies. ALDH2*2 had a negative effect on short-term prognosis, although it was unlikely to affect long-term prognosis. According to multivariable and stratified analyses, the negative effect was independent of sex, smoking habit, PD-L1 expression rate, and *EGFR* mutation. Compared with *EGFR* mutation, which has been shown to be associated with poor prognosis independent of ICI or initial ICI efficacy [30, 36], rs671 may be more strongly associated with initial ICI efficacy. However, ALDH2*2 showed no negative effect on long-term survivors, especially the first treatment line group; thus, we found that ALDH2*2 is not consistently associated with negative effects.

Several findings that support the negative impact of ALDH2*2 on the short-term prognosis of ICI therapy have been reported. Gao et al. (2018) showed that drug-induced T-cell hepatitis is suppressed by exogenous acetaldehyde. Mechanistically, aldehyde suppresses the secretion of cytokines by inhibiting the phosphatidylinositol 3-kinase (PI3K)-Akt pathway in T cells or promotes the secretion of glucocorticoids that suppresses the activation of T cells [12]. This suppression of the PI3K-Akt pathway has also been confirmed in the cardiomyocytes of *Aldh2−/−* mice, in which endogenous aldehydes accumulate in the absence of exogenous aldehyde [37]. These findings suggest that endogenous aldehyde also suppresses the PI3K-Akt pathway in T cells. The PI3K-Akt pathway is important for T cell differentiation [38] and has been shown to decrease the number of T cells in the thymus gland when activity is impaired [39, 40]. In fact, we found that the number of T cells in the peripheral blood of untreated *Aldh2−/−* mice and healthy ALDH2*2 carriers is low [25]. On the basis of these findings, we hypothesize that ALDH2*2 negatively affects the initial ICI efficacy via suppression of the PI3K-Akt pathway in T cells due to endogenous aldehyde accumulation.

Contrary to our previous finding [25], the baseline lymphocyte counts in patients in the current cohort tended to be higher in the rs671(+) group (*p* = 0.06, Table 1). Presumably, it is due to stronger antigen presentation of tumor cells, as mentioned above, associated with DNA damage due to higher aldehyde exposure than in the rs671(−) group. Baseline lymphocyte count is positively correlated with the efficacy of PD-1/PD-L1 therapy (*p* = 0.02) (Table S4), yet PFS in the first 6-month was worse in rs671(+) group than in rs671(−) group (Model 1—3, Table 4), and the finding became more obvious after adjustment of lymphocyte count (Model 4, Table 4). Thus, it is suggested that both T cell function and number are required for effective PD-1/PD-L1 therapy.

As explained above, endogenous aldehyde can also be advantageous. In the present study, the PFS ratio in the rs671(+) group decreased rapidly but was eventually higher than that of the rs671(−) group (0.21 vs. 0.27). For the first-line group, the PFS ratio after 2 years was 0.37 in the rs671(−) group and 0.40 in the rs671(+) group and was substantially higher compared with that in the other groups (the PFS ratio after 2 years was 0.18–0.23 in the rs671(−) and rs671(+) groups after the second-line treatment). Because treatment before ICI may have reduced lymphocyte in the rs671(+) group, based on general linear regression model to estimate association between log (lymphocyte count) and treatment line with adjustment for age and sex (*p* = 0.04), while no such effect was detected in rs671(−) group (*p* = 0.98), it is suggested that preventing a decrease in the number of T cells caused by pre-ICI treatment may increase the chances of obtaining a good ICI effect.

The response rate to ICIs is currently insufficient. For example, only 10–20% of patients with non-small-cell lung cancer (NSCLC) [41, 42] respond to this therapy. Therefore, optimal case selection is important. The effects of ICI are affected by the immunity of the host, the intestinal bacterial environment of the host, and tumor tissue factors [43, 44]. The PD-L1 expression level and *EGFR* mutation rate, which are tumor tissue factors, are currently used as predictors in clinical settings. In the present study, there was no association between the PFS and PD-L1 ratio, possibly due to time width between tissue evaluation and the start of the ICI. However, *EGFR* mutation was shown to be a negative predictor as previously reported, although only for rs671(−). It also has been shown that tissue infiltrating lymphocytes and tumor mutation burden can be predictors of treatment effects, although they have not been applied clinically [44–46]. The most significant limitation of these factors is that highly invasive biopsies are required. Because the microenvironment and gene mutations of tumor cells are known to fluctuate dynamically, collecting tumor tissues immediately before treatment is ideal. However, this may often be difficult due to the condition of patients and the site of lesions. Meanwhile, Hatae et al. (2020) recently showed that blood metabolites reflecting the state of intestinal bacteria and tumor-specific T cell rates are good predictors of ICI effects on NSCLC, although there are still difficulties owing to the number of tested parameters after the start of treatment [47]. Ohue et al. (2019) demonstrated that the effects of ICIs on NSCLC could be predicted by tumor antigens in blood samples collected prior to ICI initiation [HR (95% CI) of PFS in patients with antigen-positive is 0.4 (0.2 to 0.9)] [48], and its clinical application is expected. Compared with these predictors, the analysis of *ALDH2* polymorphisms has some advantages: non-invasive, inexpensive, 100% determinable, and polymorphisms do not change throughout life.

The limitations of the present study are as follows: 1) The sample size was insufficient to establish prognostic factors specific to patients with rs671(+). 2) Because the present study was limited to Japanese patients with thoracic malignancies who were mostly men, it cannot be generalized to other types of cancers and populations. 3) Because several time-dependent covariates can affect the outcome, such as adverse reactions and types and doses of second and subsequent ICIs, controlling covariates is insufficient for long-term observation. 4) The biological mechanism is not well supported.

## Conclusion

The variant allele of the *ALDH2* polymorphism rs671 was found to be a negative predictor in the early stage of PD-1/PD-L1 inhibitor treatment. However, the long-term survivor rate was the highest in the sub-group of patients with the variant allele who received an ICI as first-line treatment. The rs671 polymorphism test is expected to be a cost-effective predictor of ICI efficacy for clinical application. We need to present better personalized strategies by accumulating evidence with a larger sample size and examining the mechanism underlying the findings.

## Supplementary Information

**Additional file 1: Figure S1.** Plot of log (−log (progression-free survival ratio)) versus log (days of progression-free survival). Hazard proportionality was tested by the parallelism between the curve of rs671(−) (cases with ALDH2*1/*1) and rs671(+) (cases with ALDH22*1/*2 and ALDH2*2/*2) to examine the suitability for the Cox proportional hazard model. **Table S1.** Immune-related adverse events (IrAEs) and second or subsequent ICI doses. **Sas code.** SAS code for the Cox proportional hazards model using time-dependent explanatory variables. **Table S2.** Overall best response per RECIST Ver1.1. by *ALDH2* genotype limited to patients with non-small cell lung cancer. **Table S3.** Progression-free survival rate after the initiation of immune checkpoint inhibitors limited to patients with non-small cell lung cancer. **Table S4.** Hazaed ratio of cancer progression estimated from a 6-month observation in the model 4 in Table 4**. Figure S2.** Overall survival after the initiation of immune checkpoint inhibitor therapy. Kaplan–Meier plots were shown for patients with chest malignancies. ICI, immune checkpoint inhibitor; Rs671(−), ALDH2*1/*1 (*n* = 56), rs671(+); ALDH2*1/*2 or ALDH2*2/*2 (*n* = 49). p, *p* value for Gahan–Breslow–Wilcoxon test. **Table S5.** Hazard ratio of overall death for ALDH2*2 carriers estimated from a 6-month observation. **Table S6.** Stratified hazard ratio of overall death for ALDH2*2 carriers estimated from a 6-month observation.

## Data Availability

The datasets generated and/or analyzed during the current study are not publicly available due to a potential infringement of privacy but are available from the corresponding author on reasonable request.

## Abbreviations

ALDH2: Aldehyde dehydrogenase 2
ICIs: Immune checkpoint inhibitors
PD-1: Programmed cell death-1
PD-L1: Programmed death-ligand 1
PFS: Progression-free survival
4HNE: 4 hydroxynonenal
CR: Complete Response
PR: Partial Response
SD: Stable Disease
PD: Progressive Disease
RMST: Restricted mean survival time
HR: Hazard ratio
irAEs: Immune-related adverse events
PI3K: Phosphatidylinositol 3-kinase
NSCLC: Non-small-cell lung cancer
