# Supplementary material for: ALDH2 polymorphism rs671 is a predictor of PD-1/PD-L1 inhibitor efficacy against thoracic malignancies
Source: BMC Cancer. 2021 May 22;21:584. doi: 10.1186/s12885-021-08329-y (PMC8140463; doi:10.1186/s12885-021-08329-y)
Supplement: Supplementary file 1 — Additional file 1: Figure S1. Plot of log (−log (progression-free survival ratio)) versus log (days of progression-free survival). Hazard proportionality was tested by the parallelism between the curve of rs671(−) (cases with ALDH2*1/*1) and rs671(+) (cases with ALDH22*1/*2 and ALDH2*2/*2) to examine the suitability for the Cox proportional hazard model. Table S1. Immune-related adverse events (IrAEs) and second or subsequent ICI doses. Sas code. SAS code for the Cox proportional hazards model using time-dependent explanatory variables. Table S2. Overall best response per RECIST Ver1.1. by ALDH2 genotype limited to patients with non-small cell lung cancer. Table S3. Progression-free survival rate after the initiation of immune checkpoint inhibitors limited to patients with non-small cell lung cancer. Table S4. Hazaed ratio of cancer progression estimated from a 6-month observation in the model 4 in Table 4. Figure S2. Overall survival after the initiation of immune checkpoint inhibitor therapy. Kaplan–Meier plots were shown for patients with chest malignancies. ICI, immune checkpoint inhibitor; Rs671(−), ALDH2*1/*1 (n = 56), rs671(+); ALDH2*1/*2 or ALDH2*2/*2 (n = 49). p, p value for Gahan–Breslow–Wilcoxon test. Table S5. Hazard ratio of overall death for ALDH2*2 carriers estimated from a 6-month observation. Table S6. Stratified hazard ratio of overall death for ALDH2*2 carriers estimated from a 6-month observation. [file 12885_2021_8329_MOESM1_ESM.docx]

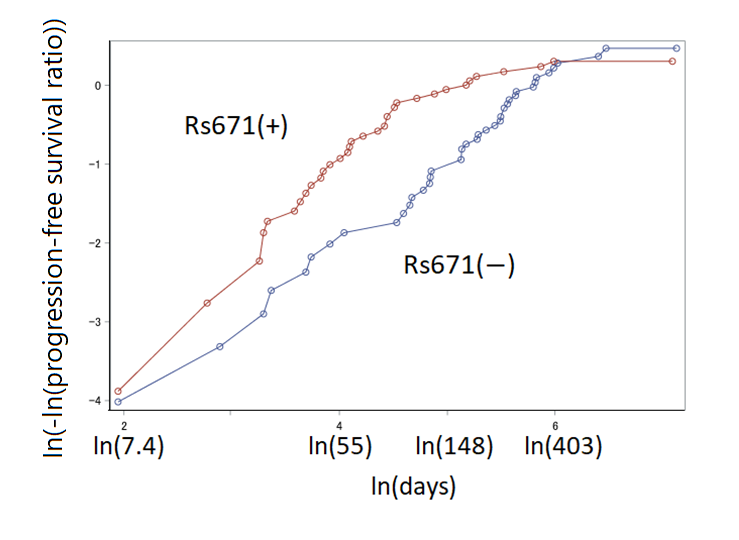


Figure S1. Plot of log (-log (progression-free survival ratio)) versus log (days of progression-free survival).

Hazard proportionality was tested by the parallelism between the curve of rs671(−) (cases with *ALDH2*1/*1*) and rs671(+) (cases with *ALDH22*1/*2* and *ALDH2*2/*2*) to examine the suitability for the Cox proportional hazard model.

| Table S1. Immune-related adverse events (IrAEs) and second or subsequent ICI doses. | | | | | | | |
| --- | --- | --- | --- | --- | --- | --- | --- |
|  | Total | | Rs671(−) | | Rs671(+) | | p |
| Diagnosis of IrAEs before disease progression* | | | | | | | |
| Within 6 months | | | | | | | |
| No | 77 | (73%) | 39 | (70%) | 38 | (78%) | 0.361 |
| Yes | 28 | (27%) | 17 | (30%) | 11 | (22%) |  |
| ―Days from ICI, median (IQR) | 77 | (50–118) | 77 | (55–123) | 65 | (16–91) | 0.359 |
| Within a year | | | | | | | |
| No | 72 | (69%) | 36 | (64%) | 36 | (73%) | 0.312 |
| Yes | 33 | (31%) | 20 | (36%) | 13 | (27%) |  |
| ―Days from ICI, median (IQR) | 80 | (55–150) | 89 | (56–159) | 80 | (46–135) | 0.461 |
| Number of ICI doses during the entire observation period | | | | | | | |
| 1–5 | 50 | (48%) | 19 | (34%) | 31 | (63%) | **0.003** |
| 6–68 | 55 | (52%) | 37 | (66%) | 18 | (37%) |  |

Rs671(−); *ALDH2*1/*1*, rs671(+); *ALDH2*1/*2* or *ALDH2*2/*2*, ICI; immune checkpoint inhibitor, IQR; interquartile range. *Prednisolone treatment for adverse events of ICIs or discontinuation of the ICI due to its adverse events. p; probability value for the Chi-squared test, Fisher’s exact test, or Wilcoxon rank-sum test. The hazard ratio (HR) of irAEs estimated by the Cox proportional hazard model also showed no difference between *ALDH2* genotypes. The HR of irAEs within 6 months for the rs671(+) group was 1.5 with a 95% confidence interval of 0.6–3.8. Similarly, the HR of irAEs within a year was 1.5 (0.6–3.6) (covariates: sex, age (continuous), Brinkman index (<100, <1000, ≥1000) (ordinal), type of first ICI, tumor histotype, TNM classification (categorical), number of lines (first, second, third, and later) (categorical), chemistry with ICI, PD-L1 positivity ratio (<1%, <50%, -100%, unassessed) (categorical), and *EGFR* mutation ((+), (-), unassessed) (categorical)).

SAS code for the Cox proportional hazards model using time-dependent explanatory variables

**proc** **phreg**;

class sex ici path stagec line chemo pdl1 egfr;

model pfsday*pfs_cens(**1**) = star2 sex age bi ici path stage line chemo pdl1 egfr aeStatus ae_t;

if (aed = . or pfsday < aed) then do;

aeStatus = 0;

ae_t = 0;

end;

else do;

aeStatus = 1;

ae_t = aed;

end;

**run**;

star2; carrier of *ALDH2*2* allele

bi; Brinkman Index

ici; type of immune checkpoint inhibitor (ICI)

path; tumor histotype

stage; TNM classification

line; Treatment line

chemo; chemotherapy with first ICI

pdl1; PD-L1 positive ratio in cancer tissue

egfr; *EGFR* mutation in cancer tissue

pfsday; period of progression-free survival

pfs_cens; censored (1) or not censored (disease progressed, 0)

aeStatus; adverse effect of ICI (0 or 1) (time-dependent variable)

aed; days from starting ICI therapy to the diagnosis of adverse effects

| Table S2. Overall best response per RECIST Ver1.1. by *ALDH2* genotype limited to patients with non-small cell lung cancer. | | | | | | | |
| --- | --- | --- | --- | --- | --- | --- | --- |
|  | Total (N = 103) | | Rs671(−) | | Rs671(+) | | p |
| Best response to immune checkpoint inhibitor |  |  |  |  |  |  |  |
| Complete response | 0 | (0%) | 0 | (0%) | 0 | (0%) | 0.0013 |
| Partial response | 32 | (31%) | 19 | (36%) | 13 | (27%) |  |
| Stable disease | 42 | (40%) | 28 | (50%) | 14 | (29%) |  |
| Progressive disease | 29 | (29%) | 7 | (14%) | 22 | (45%) |  |
| Disease control rate | 72% |  | 87% |  | 55% |  | 0.0003 |

Patients with non-small cell lung cancer (excluding mesothelioma) were subjected. Rs671(−); *ALDH2*1/*1*, rs671(+); *ALDH2*1/*2* or *ALDH2*2/*2*, disease control rate; (all − progressive disease)/all, p; probability value for Chi-squared test.

| Table S3. Progression-free survival rate after the initiation of immune checkpoint inhibitors limited to patients with non-small cell lung cancer. | | |
| --- | --- | --- |
| Observation period | Restricted mean survival time | |
|  | Rs671(−) | Rs671(+) |
| 0–6 months | 0.82 | 0.58 |
| 6–12 months | 0.46 | 0.31 |
| 12–24 months | 0.24 | 0.26 |
| 24–36 months | 0.19 | 0.26 |
| Patients with non-small cell lung cancer (excluding mesothelioma) were subjected. Rs671(−); *ALDH2*1/*1*, rs671(+); *ALDH2*1/*2* or *ALDH2*2/*2*. | | |

| Table S4. Hazaed ratio of cancer progression estimated from a 6-month observation in the model 4 in Table 4. | | | | |
| --- | --- | --- | --- | --- |
| Variables | Hazard ratio | 95% confidence interval | | p |
| Rs671 |  |  |  |  |
| *ALDH2*1/*1* | 1.00 | Reference |  |  |
| *ALDH2*1/*2* or *ALDH2*2/*2* | 5.42 | 2.65 | 11.1 | <.0001 |
| Sex |  |  |  |  |
| Male | 1.00 | Reference |  |  |
| Female | 4.24 | 1.58 | 11.4 | 0.0042 |
| Age |  |  |  |  |
| Per one year | 1.00 | 0.96 | 1.04 | 0.8876 |
| Brinkman Index |  |  |  |  |
| Per one category (<100, <1000, ≥1000) | 1.59 | 0.93 | 2.73 | 0.0912 |
| Type of ICI (first dose) |  |  |  |  |
| Pembrolizumab | 1.00 | Reference |  |  |
| Atezolizumab | 1.32 | 0.44 | 3.94 | 0.6204 |
| Nivolumab | 0.74 | 0.24 | 2.32 | 0.6031 |
| Tumor histotype |  |  |  |  |
| Squamous cell carcinoma | 1.00 | Reference |  |  |
| Adenocarcinoma | 2.38 | 0.86 | 6.59 | 0.0938 |
| Pleomorphic carcinoma | 1.15 | 0.19 | 7.08 | 0.8812 |
| Mesothelioma | 1.94 | 0.17 | 22.7 | 0.5996 |
| Other† | 7.18 | 1.31 | 39.3 | 0.023 |
| TNM classification |  |  |  |  |
| Stage III | 1.00 | Reference |  |  |
| Stage IV | 1.51 | 0.59 | 3.86 | 0.3885 |
| Unknown | 1.86 | 0.64 | 5.38 | 0.2536 |
| Treatment line |  |  |  |  |
| First-line | 0.45 | 0.13 | 1.55 | 0.2046 |
| Second-line | 0.95 | 0.37 | 2.49 | 0.9231 |
| Third-line and later | 1.00 | Reference |  |  |
| Chemotherapy with first ICI |  |  |  |  |
| No | 1.00 | Reference |  |  |
| Yes | 0.23 | 0.02 | 2.12 | 0.1923 |
| PD-L1 (+) ratio in cancer tissue |  |  |  |  |
| <1％ | 1.00 | Reference |  |  |
| <50％ | 2.70 | 0.89 | 8.18 | 0.0798 |
| ≥50% | 2.11 | 0.60 | 7.35 | 0.2421 |
| Unassessed | 2.49 | 0.73 | 8.45 | 0.143 |
| *EGFR* mutation in cancer tissue |  |  |  |  |
| (−) | 1.00 | Reference |  |  |
| (+) | 2.63 | 0.80 | 8.67 | 0.1125 |
| Unassessed | 4.72 | 1.22 | 18.22 | 0.0243 |
| Neutrophil count in peripheral blood |  |  |  |  |
| Per 2.72x | 5.09 | 2.51 | 10.32 | <.0001 |
| Lymphocyte count in peripheral blood |  |  |  |  |
| Per 2.72x | 0.45 | 0.23 | 0.89 | 0.0209 |
| All variables in the model 4 in Table 4 are shown. ICI; immune checkpoint inhibitor, PD-L1; programmed death-ligand 1, *EGFR*; epidermal growth factor receptor. † includes combined small cell lung carcinoma, adenosquamous carcinoma of the lung, and non-small-cell lung cancer-not otherwise specified. | | | | |
|  |  |  |  |  |
|  |  |  |  |  |


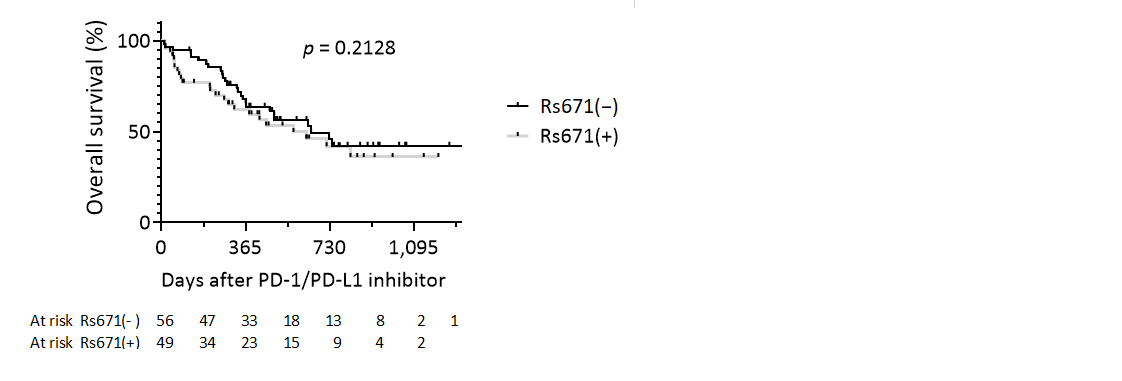


Figure S2. Overall survival after the initiation of immune checkpoint inhibitor therapy.

Kaplan–Meier plots were shown for patients with chest malignancies. ICI, immune checkpoint inhibitor; Rs671(−), *ALDH2*1/*1* (n = 56), rs671(+); *ALDH2*1/*2* or *ALDH2*2/*2* (n = 49). p, p value for Gahan–Breslow–Wilcoxon test.

Table S5. Hazard ratio of overall death for *ALDH2*2* carriers estimated from a 6-month observation.

|  | Rs671 |  | HR | 95％ CI | p | AIC |
| --- | --- | --- | --- | --- | --- | --- |
| Model 1 | (−) |  | 1.00 | (reference) |  | 166 |
|  | (+) |  | 3.33 | (1.10–10.1) | 0.034 |  |
| Model 2 | (−) |  | 1.00 | (reference) |  | 170 |
|  | (+) |  | 4.55 | (1.34–15.4) | 0.015 |  |
| Model 3 | (−) |  | 1.00 | (reference) |  | 167 |
|  | (+) |  | 3.95 | (1.17–13.3) | 0.027 |  |
| Model 4 | (−) |  | 1.00 | (reference) |  | 152 |
|  | (+) |  | 13.6 | (2.38-77.8) | 0.003 |  |
| Model 5 | (−) |  | 1.00 | (reference) |  | 142 |
|  | (+) |  | 16.8 | (2.2-127) | 0.006 |  |

Rs671(−); *ALDH2*1/*1*, rs671(+); *ALDH2*1/*2* or *ALDH2*2/*2,* HR; hazard ratio by Cox proportional hazard model, CI; confidence interval, AIC; Akaike’s Information Criterion.

Model 1: adjusted for sex, age (continuous), Brinkman Index (<100, <1000, ≥1000) (ordinal), type of first immune checkpoint inhibitor (ICI), tumor histotype, TNM classification (categorical), number of lines (first, second, third, and later) (categorical), and chemotherapy with ICI.

Model 2: adjusted for the covariates in model 1 and the PD-L1 positivity ratio (<1%, <50%, -100%, unassessed).

Model 3: adjusted for the covariates in model 2 and *EGFR* mutation ((+), (-), unassessed).

Model 4: adjusted for the covariates in model 3, log(neutrophil count in peripheral blood) and log(lymphocyte count in peripheral blood).

Model 5: adjusted for the covariates in model 4 and time-dependent variables (the presence or absence of immune-related adverse events and timing of onset).

| Table S6. Stratified hazard ratio of overall death for *ALDH2*2* carriers estimated from a 6-month observation. | | | |
| --- | --- | --- | --- |
|  | HR | 95% CI | p |
| Sex |  |  |  |
| Male (N = 89) | 3367 | 7.36—>9999 | *0.010* |
| Female (N = 16) | Non-estimable | | |
| Age |  |  |  |
| <70 (N = 55) | Non-estimable | | |
| ≥70 (N = 50) | 38.2 | 0.94—1548 | *0.054* |
| Brinkman Index |  |  |  |
| <1000 (N = 53) | Non-estimable | | |
| ≥1000 (N = 52) | Non-estimable | | |
| Type of immune checkpoint inhibitor |  |  |  |
| Pembrolizumab (N = 45) | Non-estimable | | |
| Nivolumab and Atezolizumab (N = 60) | 18.7 | 1.58—220 | *0.020* |
| Tumor histotype |  |  |  |
| Adenocarcinoma (N = 64) | 5.03 | 0.9—28.2 | *0.066* |
| Others (N = 41) | Non-estimable | | |
| TNM classification |  |  |  |
| Stage III (N = 25) | Non-estimable | | |
| Stage IV (N = 51) | Non-estimable | | |
| Treatment line |  |  |  |
| First-line (N = 41) |  | | |
| Second-line and later (N = 64) | 30.0 | 2.50—360 | *0.007* |
| Chemotherapy with first ICI |  |  |  |
| Yes (N = 6) | Non-estimable | | |
| No (N = 99) | 13.6 | 2.38—77.8 | *0.003* |
| PD-L1 (+) ratio in cancer tissue |  |  |  |
| <50% (N = 42) | Non-estimable | | |
| ≥50% (N = 48) |  | | |
| *EGFR* mutation in cancer tissue |  |  |  |
| (+) (N = 8) | Non-estimable | | |
| (−) (N = 81) | 270 | 3.18—22973 | *0.014* |
| Neutrophil count in peripheral blood |  |  |  |
| <4000/μL (N = 53) | Non-estimable | | |
| ≥4000/μL (N = 52) | Non-estimable | | |
| Lymphocyte count in peripheral blood |  |  |  |
| <1300/μL (N = 52) | Non-estimable | | |
| ≥1300/μL (N = 53) | Non-estimable | | |
| Reference = *ALDH2*1/*1* carriers. HR; hazard ratio by Cox proportional hazard model adjusted for covariates used in Model 4 in table S5, CI; confidence interval, PD-L1; programmed death-ligand 1, *EGFR*; epidermal growth factor receptor. Cases with unknown TNM classification (N = 29), unknown PD-L1 (+) ratio in cancer tissue (N = 15), and unknown *EGFR* mutation in cancer tissue (N = 16) were excluded. | | | |
|  |  |  |  |
|  |  |  |  |
|  |  |  |  |
|  |  |  |  |
